# Social Marketing for Health Equity: Promoting Preventive Health Behavior Among Women in Rural Communities

**DOI:** 10.3390/ijerph23050584

**Published:** 2026-04-30

**Authors:** Kamel Mouloudj, Sarah Ali Saeed Alameri, Marian A. Evans, Alaa Abdulkareem Ghaleb Almado, Basheer Ismail Mahmoud, Dachel Martínez Asanza

**Affiliations:** 1 College of Economic, University of Medea, Medea 26000, Algeria; 2 Department of Business Administration, College of Administration and Economics, University of Baghdad, Baghdad 10071, Iraq; dr.sarah_ali@coadec.uobaghdad.edu.iq (S.A.S.A.); alaa.abdulkareem@coadec.uobaghdad.edu.iq (A.A.G.A.); basheer@mracpc.uobaghdad.edu.iq (B.I.M.); 3 Department of Public Health, College of Health and Human Services, Southern Connecticut State University, New Haven, CT 06515, USA; evansm7@southernct.edu; 4 Department of Scientific Technical Results Management, National School of Public Health (ENSAP), Havana Medical Sciences University, Havana 10800, Cuba; dachelmtnez@infomed.sld.cu

**Keywords:** preventive health behaviors, health literacy, health equity, health policy, women in rural communities, perceived healthcare discrimination

## Abstract

**Highlights:**

**Public health relevance—How does this work relate to a public health issue?**

- This study examines the factors associated with the intentions of women in rural communities to adopt preventive health behaviors using an extended Theory of Planned Behavior (TPB) framework.
- It addresses public health challenges related to limited access to healthcare services, health awareness, and preventive practices among women in rural communities in Algeria.

**Public health significance—Why is this work of significance to public health?**

- The findings show that attitude, subjective norms, “perceived behavioral control” (PBC), and health literacy are significantly associated with preventive health intentions among women in rural communities.
- The extended model explains a substantial proportion of behavioral intention (57.5%), highlighting the importance of psychosocial and informational factors in understanding preventive health intentions.

**Public health implications—What are the key implications or messages for practitioners, policy makers and/or researchers in public health?**

- Public health interventions should focus on strengthening positive attitudes, enhancing social support, improving PBC, and increasing health literacy to support preventive health intentions among women in rural communities.
- Policymakers and healthcare stakeholders may consider designing targeted health communication strategies and community-based outreach programs to improve health awareness and reduce health disparities in rural settings.

**Abstract:**

Preventive health behaviors play a critical role in reducing disease risks and improving public health outcomes, particularly among vulnerable populations such as women in rural communities. However, limited research has explored the determinants of intentions to adopt preventive health behaviors in developing contexts among women in rural communities. This study applies and extends the Theory of Planned Behavior (TPB) to examine these determinants in Algeria. A cross-sectional study was conducted using convenience sampling among 205 women in rural communities aged 20–60 years across five Algerian cities. Data were collected through a self-administered questionnaire and analyzed using hierarchical multiple regression. The results indicate that attitude, subjective norms, and perceived behavioral control have significant positive effects on behavioral intention. The inclusion of health literacy significantly enhances the model’s explanatory power, with higher literacy associated with stronger intentions. In contrast, perceived healthcare discrimination does not have a statistically significant effect. The extended model explains 57.5% of the variance in behavioral intention. These findings underscore the importance of psychosocial and informational factors in shaping preventive health intentions and support the extension of TPB in this context. They also provide practical implications for policymakers and healthcare practitioners to design targeted social marketing interventions aimed at improving preventive health behaviors and reducing health disparities among women in rural communities.

## 1. Introduction

Promoting preventive health behaviors among women has received increasing attention in recent years, particularly among women living in rural areas [1,2]. Beyond reducing exposure to potential health risks, these behaviors improve quality of life and lower healthcare costs [3]. There is no doubt that unhealthy behaviors increase the likelihood of developing various diseases (such as diabetes, hypertension, and cardiovascular diseases) [1,4,5].

In practice, women in rural communities in many developing countries frequently face structural discrimination and social norms that hinder their ability to seek and obtain adequate healthcare [6,7]. Limited availability of healthcare facilities, combined with cultural beliefs, low awareness, and restrictive gender roles, creates an environment that does not support the adoption of preventive health behaviors [8,9]. According to numerous studies, rural populations often experience higher morbidity and mortality rates from preventable diseases, underscoring the urgent need for targeted interventions that promote health equity and improve access to healthcare services [10]. In Algeria, as in many developing countries, women in rural communities encounter significant barriers, including limited access to healthcare services, poverty, low health awareness and literacy, weak and insufficient healthcare provision, and policy and institutional constraints, such as the lack of policies specifically designed to address the needs of rural populations [6,7,11].

In the Algerian context specifically, these challenges are exacerbated by geographical disparities in healthcare infrastructure due to the country’s vast size (2.381 million km^2^), the unequal distribution of medical resources, and deeply entrenched social and cultural norms that influence women’s healthcare-seeking behavior [12,13]. Despite public health efforts, preventive healthcare practices remain insufficiently implemented among women in rural communities, highlighting the urgent need for context-specific research that goes beyond general evidence drawn from other developing regions.

Within this framework, social marketing activities represent an appropriate and proven approach for modifying and changing various negative behaviors, such as smoking, drug addiction, and violence [14,15,16]. Indeed, the intersection between social marketing and health equity has attracted growing scholarly interest, particularly in rural contexts (e.g., [17]). This focus is especially critical in developing countries like Algeria, where women in rural communities often face substantial barriers to accessing healthcare and adopting preventive health behaviors [6,11]. Social marketing, defined as “an approach used to develop activities that are aimed at changing or maintaining people’s behaviour for the benefit of individuals and society as a whole” [17] (p. 2), provides a valuable framework for addressing such disparities [14]. By encouraging preventive health behaviors, social marketing communications can empower women in rural communities, enhance health awareness, and ultimately contribute to more equitable health outcomes [18,19].

In a related vein, the “Theory of Planned Behavior” (TPB) offers a robust framework for understanding the psychological determinants of health behaviors. Developed by Ajzen [20], the theory posits that individual behavior is shaped by three principal components: “attitudes toward the behavior”, “subjective norms”, and “perceived behavioral control” (PBC). These elements interact to form behavioral intentions, which are critical for the adoption of preventive health practices [8,21,22]. However, despite its explanatory power, many scholars have extended the TPB by incorporating additional factors to enhance its predictive capacity. For example, Jooyandeh et al. [23] expanded the TPB to include perceived risk and social media variables. Similarly, Rajeh [24] extended it by integrating health knowledge. Other studies have combined the TPB with the “Health Belief Model”(HBM) to predict tourists’ health risk prevention behaviors [25,26,27], while Xu et al. [28] integrated the TPB with the self-efficacy to explore preventive health screening behaviors. Accordingly, incorporating health literacy into this model is essential, as it enables individuals to access health information and make informed decisions regarding their health [10,29,30]. Moreover, health literacy empowers women to challenge prevailing norms and advocate for their health needs [31].

Nevertheless, the broader context in which these women operate cannot be overlooked. Perceived discrimination in healthcare settings emerges as a significant barrier that may negatively influence their intentions to adopt preventive health behaviors [32,33,34]. Research indicates that when individuals perceive discrimination in healthcare facilities, their trust in healthcare providers may decline, leading to reduced healthcare-seeking behavior [35,36,37,38,39]. Therefore, addressing perceived discrimination is crucial in designing effective communication and health equity strategies that are responsive to the needs of vulnerable groups [40,41], such as women in rural communities.

Despite growing international evidence on TPB-based models and their applications in predicting health behaviors, investigations into these relationships in the Algerian context [11,12,13], particularly among women in rural communities, remain limited. Furthermore, previous studies have rarely integrated health awareness and perceived discrimination in healthcare within the TPB framework, despite their importance in shaping health decision-making in resource-limited and socially unequal settings. Bridging this gap is therefore crucial, as these factors embody the informational and structural dimensions of health equity, which are often overlooked when applying behavioral theories in developing contexts. Accordingly, this study aims to address this gap by extending planned behavior theory to include health culture and perceived discrimination in healthcare, with the goal of better understanding preventive health behavior intentions among women in rural communities in Algeria. Specifically, the study aims to examine the relationships between attitudes, subjective norms, PBC, health literacy, and perceived discrimination, and behavioral intentions.

The findings are expected to enrich social marketing campaigns by not only addressing the specific needs of women in rural communities but also tackling broader social determinants of health [15,18]. By enhancing health literacy and confronting discriminatory practices within healthcare systems, a supportive environment can be fostered that encourages the adoption of preventive health behaviors. Effective health communication campaigns grounded in social marketing principles can help bridge the gap between knowledge and practice, ultimately improving health outcomes and promoting equity among women in rural communities.

## 2. Literature Review and Hypotheses Development

### 2.1. TPB in Healthcare

Ausserhofer et al. [8] noted that preventive health behavior is often explained using theoretical models that describe how individuals make decisions regarding prevention and self-care. They further indicated that the TPB is among the most widely applied frameworks in this field. The TPB, developed by Ajzen [20], is a widely recognized framework for understanding behavioral intentions and actions across various domains, including healthcare. The theory proposes that an individual’s behavior is primarily influenced by attitudes toward the behavior, subjective norms, and perceived behavioral control. In healthcare contexts, TPB has been extensively used to examine how these factors influence health-related behaviors such as adherence to medical advice, avoidance of unhealthy behaviors, engagement in preventive care, and lifestyle or behavioral changes [10,22,42]. Research by Dangaiso et al. [21] and Park and Oh [27] found that behavioral intention is a strong predictor of preventive health behaviors, indicating that understanding individuals’ intentions can help design effective interventions aimed at promoting preventive health practices, while also acknowledging the potential barriers that may limit some individuals’ ability to perform the desired behaviors.

Within this framework, previous studies have shown that positive attitudes toward health behaviors, often associated with knowledge, awareness of consequences, and beliefs, can significantly increase the likelihood of engaging in preventive healthcare practices [10,43]. In addition, subjective norms, which refer to perceived social pressure and the influence of reference groups, play a crucial role. For example, women may be more likely to adopt certain health behaviors when they receive encouragement and support from family members and peers [22]. Finally, PBC reflects individuals’ perceptions of the barriers or facilitators that may affect their ability to perform preventive health behaviors [21]. For women in rural communities, such barriers may include transportation difficulties, financial constraints, or limited access to healthcare facilities [6]. Therefore, integrating TPB into health promotion strategies enables practitioners and stakeholders to design targeted interventions that address these specific influences, ultimately improving health behaviors and healthcare outcomes within underserved and marginalized communities.

### 2.2. Preventive Health Behavior Among Women in Rural Communities

Preventive health behaviors refer to actions undertaken to prevent diseases or health problems before they occur, and they are particularly important for vulnerable populations [42], such as women in rural communities. These behaviors include regular health screenings, vaccinations, and lifestyle modifications such as physical exercise and healthy nutrition [44]. In this context, Satoh and Sato [19] indicated that decisions to engage in preventive health behaviors are closely related to the perceived likelihood of illness or death. When individuals perceive their health as being at risk, they are more motivated to take proactive measures to protect themselves [31]. Furthermore, Ausserhofer et al. [8] noted that preventive health behaviors are influenced not only by personal knowledge or demographic factors (such as age and gender), but also by a combination of individual capabilities (e.g., self-confidence) and contextual factors (e.g., social isolation and trust in health information). In practice, women’s preventive health behaviors are shaped by multiple determinants, including individual, economic, social, and cultural factors, as well as social marketing activities [1,4,17].

In the context of preventive behaviors during the COVID-19 pandemic, Sánchez-Arenas et al. [30] found that women and older adults were more likely to adopt preventive measures. Their study also revealed that such behaviors were associated with engagement in physical activity, improved health literacy, access to information sources, perceived risk, and perceived effectiveness of preventive measures. However, research suggests that women in rural communities are often less likely to adopt preventive health behaviors compared with their urban counterparts. This disparity is largely attributed to structural inequalities and systemic barriers, such as limited access to healthcare services, lack of information, low awareness of health practices and consequences, cultural constraints, and financial costs [1,19,43,44]. Additionally, Satoh and Sato [19] reported that women who engage in unhealthy habits—such as smoking, alcohol consumption, and poor dietary patterns—are less likely to adopt preventive health behaviors than women who maintain healthier lifestyles.

In rural settings, women may often prioritize the immediate needs of their families over their own health, reflecting a form of personal sacrifice that can lead to delays or missed opportunities for seeking healthcare, potentially resulting in adverse health outcomes. Moreover, health literacy plays a crucial role in understanding the importance of preventive measures. Women with higher levels of health literacy are generally more inclined to adopt proactive health behaviors [10,17,45]. Therefore, addressing these disparities is essential for improving health outcomes among women in rural communities. Social marketing strategies, combined with the TPB framework, may provide valuable insights for designing effective interventions aimed at enhancing awareness, correcting misconceptions, and empowering women to manage their health, ultimately contributing to greater health equity and improved public health outcomes [15,18].

### 2.3. Hypotheses Development

#### 2.3.1. Attitudes Toward Preventive Health Behaviors

Attitude was described as “the degree to which a person has a favorable or unfavorable evaluation or appraisal of the behavior in question” [20] (p. 188). Attitudes play a crucial role in shaping behavioral intentions within the framework of the TPB. When individuals hold a positive attitude toward preventive health behaviors, their likelihood of intending to engage in these behaviors increases [21,46]. Previous studies have shown that attitudes toward preventive health behaviors are influenced by factors such as awareness, knowledge, trust in health information, and social environments [8,47,48,49]. Research on preventive behaviors has demonstrated associations between attitudes, behavioral intentions, and actual engagement [19,26,28,42]. Furthermore, Fatima et al. [1] reported that negative attitudes reduce pregnant women’s willingness to adopt healthy behaviors, while Huang et al. [25] found that attitudes predict preventive health behaviors among tourists. For women in rural communities, positive attitudes toward preventive health measures, such as regular check-ups, vaccinations, or healthy eating, can be fostered through effective social media and health awareness campaigns that highlight the benefits of these behaviors [9,23]. However, Park and Oh [27] found that attitudes did not significantly predict intentions or preventive health behaviors related to COVID-19. Based on this evidence, the following hypothesis is proposed:

**H1.**  *Attitudes toward preventive health behaviors positively associated with intentions to engage in these behaviors among women in rural communities*.

#### 2.3.2. Subjective Norms

Ajzen [20] (p. 188) defined subjective norms as “the perceived social pressure to perform or not to perform the behavior.” Subjective norms encompass the perceived social pressures to engage in a behavior, which significantly influence individuals’ behavioral intentions [21]. In the context of women in rural communities, family and community expectations often shape health-related decisions. When women perceive that their peers and family members value preventive health behaviors, they are more likely to intend to engage in these behaviors themselves [20]. This is particularly important in collectivist cultures, where social approval can serve as a strong motivating factor. Interventions aimed at strengthening supportive social networks and highlighting preventive health champions within the community can enhance subjective norms [23,47]. Empirically, subjective norms are associated with increased health awareness and knowledge, which in turn strengthen and improve intentions and positive preventive health behaviors (e.g., [24,26,27,28,42,43,46]). However, Huang et al. [25] suggested that subjective norms are often an imprecise predictor of preventive health behaviors. Furthermore, the quality of information conveyed through subjective norms is expected to play an important role in shaping women’s attitudes and intentions toward adopting preventive health practices [50]. Based on this reasoning, the following hypothesis is proposed:

**H2.**  *Subjective norms positively associated with intentions to engage in preventive health behaviors among women in rural communities*.

#### 2.3.3. Perceived Behavioral Control

According to the TPB, PBC is defined as “people’s perception of the ease or difficulty of performing the behavior of interest” [20] (p. 183). This construct reflects a person’s belief in their ability to perform a given behavior, taking into account potential barriers and facilitators they may encounter [22]. In healthcare settings, particularly for women in rural communities, perceived barriers, such as transportation, cost, limited access to facilities, lack of knowledge, and geographic distance, can significantly influence their intentions to adopt preventive health behaviors [20,29,51]. When women perceive that they have the necessary resources and support to overcome these barriers, their confidence in performing health-promoting behaviors increases [1,2,19]. Huang et al. [25] also found that self-efficacy predicts preventive health behaviors. Research consistently shows that higher levels of PBC are associated with stronger intentions to engage in health behaviors [8,21,26,27,28,42,46]. However, Lu and Shi [52] reported that individuals’ belief in their ability to control health risks may, in some cases, reduce their engagement in preventive behaviors aimed at avoiding those risks. Based on this evidence, the following hypothesis is proposed:

**H3.**  *PBC positively associated with intentions to adopt preventive health behaviors among women in rural communities*.

#### 2.3.4. Health Literacy

Health literacy refers to “the ability to access, understand, appraise, and apply health information for making appropriate health decisions” [53] (p. 1). It is considered a crucial cognitive resource, as it enables individuals to access, understand, and effectively use health information [30]. Health literacy also helps women identify different types of diseases, recognize risk factors, understand their modes of transmission, and learn the most effective ways to prevent them [10,31,54]. Higher levels of health literacy are associated with improved health outcomes and greater engagement in preventive health behaviors [2,8,19,45,54]. Fatima et al. [1] reported that limited knowledge and low awareness reduce women’s engagement in health-promoting behaviors. Gardiner et al. [5] also noted that due to time constraints, physicians may not always be able to provide patients with sufficient health information.

In the context of the COVID-19 pandemic, Lu and Shi [52] explained that health knowledge and skills may provide individuals with a greater sense of control over health risks, which could sometimes negatively influence risk-avoidance behaviors. For women in rural communities, health literacy can empower them to navigate the complexities of healthcare systems, understand the importance of preventive measures, and make informed decisions about their health. Njoku et al. [55] reported low levels of disease-related knowledge and limited access to treatment among rural populations. Moreover, Liu et al. [56] found that educational interventions (both face-to-face and digital) had a significant positive impact on women’s health literacy, attitudes, and behaviors. However, Rajeh [24] found that health knowledge did not predict intentions to engage in oral health preventive behaviors. Based on this reasoning, the following hypothesis is proposed:

**H4.**  *Health literacy positively associated with intentions to engage in preventive health behaviors among women in rural communities*.

#### 2.3.5. Perceived Healthcare Discrimination

Perceived healthcare discrimination refers to the belief that an individual has been treated unfairly within healthcare settings due to personal characteristics such as religion, race, gender, age, or socioeconomic status [32,57,58]. Such perceptions may erode trust in healthcare providers and discourage individuals from seeking healthcare services [36,37,39,49]. Im and Tefera [35] found that perceived discrimination was the strongest factor contributing to barriers in accessing healthcare services, with some women from specific backgrounds reporting experiences of discrimination. Similarly, Klein et al. [59] indicated that perceived discrimination is closely associated with treatment non-adherence (e.g., failure to follow medical advice, missing follow-up appointments, not filling prescriptions, or not obtaining prescribed medications), which negatively affects treatment outcomes.

For women in rural communities, experiences of discrimination may significantly hinder their willingness to adopt preventive health behaviors. When women feel marginalized, unfairly treated, or disrespected within healthcare organizations, they may avoid seeking necessary care due to fears of further discrimination, marginalization, or inadequate treatment [33,36,55,58,60,61]. Accordingly, addressing perceived healthcare discrimination is essential to fostering a supportive healthcare environment in which all women feel valued and empowered to engage in preventive health measures [34,39,40]. However, Cruz-Riveros et al. [62] found that “perceived racial and ethnic discrimination” had a positive effect on individuals’ intentions to engage in healthcare services. Therefore, higher levels of perceived healthcare discrimination are expected to negatively influence intentions to engage in preventive health behaviors among women in rural communities.

**H5.**  *Perceived healthcare discrimination negatively associated with intentions to engage in preventive health behaviors among women in rural communities*.

## 3. Materials and Methods

### 3.1. Participants

The study population included women in rural communities aged between 20 and 60 years residing in rural areas within the administrative regions of Blida, Médéa, Bouira, Tizi Ouzou, and Boumerdès. In this study, “women in rural communities” refers to women living in geographically rural communities characterized by limited access to healthcare services, lower population density, and fewer infrastructural resources compared to urban centers. Due to the limited accessibility of participants, a convenience sampling method was adopted, as it was considered the most appropriate approach to reach the target group [13]. Although convenience sampling was employed, efforts were made to improve sample diversity and reduce potential bias by collecting data from multiple rural areas across different regions and from various healthcare-related settings.

Eligibility criteria included women aged 20–60 years residing in rural areas and able to read and understand the questionnaire. Participation was not restricted based on health status; therefore, pregnant women and women with chronic diseases were not specifically excluded or targeted, as the study focused on general preventive health behaviors rather than specific medical conditions. Individuals who were unable to read or complete the questionnaire were excluded from the study.

A total of 280 women were initially selected for participation. The questionnaire was distributed between early October and the end of November 2025. From an ethical perspective, this study complies with the institutional guidelines and the applicable national regulations in Algeria, which stipulate that non-interventional survey-based research involving anonymous data does not require formal ethics committee approval. Participation was entirely voluntary and anonymous, and no incentives were offered. Verbal informed consent was obtained from all participants, which is consistent with local ethical standards for minimal-risk research involving non-identifiable data. The study posed no foreseeable physical, psychological, or social risks to participants.

Participants were approached in person, primarily by one of the authors, at various data collection sites, including medical clinics, pharmacies, screening centers, and medical analysis laboratories. In addition, some questionnaires were distributed with the assistance of staff members working in the healthcare facilities where the study was conducted. A total of 218 questionnaires were returned. After screening the responses, 13 questionnaires were excluded due to incomplete information. Consequently, the final sample consisted of 205 valid responses, which were used for the subsequent statistical analyses.

### 3.2. Research Instrument

This study adopted a quantitative research approach, in which primary data were collected using a self-administered questionnaire. The questionnaire was divided into two main sections. The first section included demographic questions, such as marital status, age, education, and occupation. The second section consisted of 18 items related to the study variables (three items for each construct). These items were adapted from previous studies and modified to fit the context of the present research. Respondents’ perceptions were measured using a five-point Likert scale ranging from 1 = “Strongly disagree” to 5 = “Strongly agree.” In this context, attitudes toward preventive health behaviors were measured using a scale adapted from Bouarar et al. [12] and Huang et al. [25]. Subjective norms were measured using scales from Brouwer and Mosack [63] and Park and Oh [27], while PBC was measured using the scale developed by Hüsser et al. [26]. In addition, health literacy was measured using the scale proposed by Ausserhofer et al. [8], and perceived healthcare discrimination was measured using the scale developed by Hausmann et al. [40] and Krieger et al. [41]. Finally, behavioral intention was measured using a scale adapted from Brouwer and Mosack [63] and Hüsser et al. [26].

On average, completing the questionnaire required approximately 12–15 min. A pilot study was conducted with 25 respondents to assess the clarity and reliability of the questionnaire items. Furthermore, two experts, one in the field of public health and the other in social marketing, were invited to review the questionnaire to ensure content validity and contextual appropriateness. Based on their feedback, several items were refined to improve clarity and relevance. The questionnaire was originally designed in English and subsequently translated into Arabic, the participants’ native language, by two experts to ensure linguistic accuracy. Only participants who were able to read and understand the questionnaire were included in the study. During data collection, the presence of the researcher allowed participants to request clarification when needed, thereby supporting comprehension of the questionnaire items. The measurement items used in the final version of the questionnaire are presented in Appendix A.

### 3.3. Statistical Analysis

The primary data were processed using SPSS 26 (“Version 26.0, IBM, Armonk, NY, USA”). Descriptive statistics, including means and standard deviations, were computed. Skewness and kurtosis were examined to assess the normality of the data distribution. Cronbach’s alpha was used to evaluate the reliability of the measures. Furthermore, tolerance and “Variance Inflation Factor” (VIF) tests were conducted to check for multicollinearity. Pearson correlation analysis was performed to examine the relationships between independent and dependent variables. Subsequently, hierarchical multiple regression was conducted to test the hypotheses and explore the feasibility of extending the TPB, using the coefficient of determination (R^2^) as an indicator of explanatory power.

## 4. Results

### 4.1. Sample Profile

As shown in Table 1, the demographic profile of the respondents indicates that married women constitute the majority of the sample (50.73%), followed by single participants (30.73%), while 13.17% preferred not to disclose their marital status and 5.37% were divorced. In terms of age distribution, the largest proportion of respondents falls within the 31–40 age group (34.15%), followed by 41–50 years (27.80%), while 20–30 years (20.49%) and 51–60 years (17.56%) represent smaller segments. Regarding educational attainment, a substantial proportion of respondents reported high school education or below (39.51%), while 32.20% held a bachelor’s degree, 21.95% a master’s degree, and 6.34% a doctorate, reflecting a relatively diverse educational background. With respect to employment status, 31.71% of the respondents were employed in the public or private sector, 30.24% were unemployed, 19.51% were self-employed, and 18.54% were students.

### 4.2. Descriptive Analysis

Table 2 presents the descriptive statistics of the study constructs, including the mean, standard deviation, Cronbach’s alpha, skewness, and kurtosis values. The reliability analysis indicates that all constructs demonstrate acceptable internal consistency, with Cronbach’s alpha values ranging from 0.701 to 0.899, exceeding the recommended threshold of 0.70 [64]. This confirms the reliability of the measurement scales used in the study.

The mean values indicate varying levels of agreement among respondents across the constructs. Health literacy recorded the highest mean value (M = 4.27), suggesting that the participating women in rural communities generally reported a relatively high level of awareness and understanding of health-related information. Behavioral intention also showed a relatively high mean (M = 3.99), indicating that respondents tend to express a strong intention to adopt preventive health behaviors. Similarly, PBC exhibited a moderately high mean (M = 3.69), suggesting that participants generally perceive themselves as having a certain degree of control over engaging in preventive health practices. In contrast, attitude toward preventive health behaviors shows a moderately positive level (M = 3.56), reflecting generally favorable perceptions among respondents. Perceived healthcare discrimination demonstrates a moderate level (M = 3.14), indicating that some respondents perceive the presence of discrimination in healthcare contexts, although the perception is not particularly strong. Meanwhile, subjective norms report the lowest mean value (M = 2.82).

With regard to data distribution, the skewness values range between −1.419 and 0.276, which falls within the acceptable threshold of ±2, indicating that the data are not severely skewed. Similarly, the kurtosis values range between −0.583 and 3.625, remaining well within the recommended range of ±7. These results suggest that the distribution of the study variables does not deviate significantly from normality [11].

Table 3 presents the Pearson correlation coefficients among the study constructs, highlighting the relationships between independent variables and behavioral intentions. The results indicate that attitude (r = 0.545, *p* < 0.01), subjective norms (r = 0.459, *p* < 0.01), PBC (r = 0.582, *p* < 0.01), and health literacy (r = 0.568, *p* < 0.01) are all positively and significantly correlated with behavioral intention, suggesting that higher levels of these factors are associated with stronger intentions among women in rural communities to engage in preventive health behaviors. Among these variables, PBC and health literacy show the strongest correlations with intention, indicating their relatively greater importance in shaping behavioral intentions. In contrast, perceived healthcare discrimination shows a very weak and non-significant correlation with behavioral intention (r = 0.017), suggesting that it may not play a meaningful role in influencing preventive health intentions in this sample.

### 4.3. Hypothesis Testing

Table 4 presents the results of the hierarchical multiple regression analysis, which was conducted to examine the effects of the independent variables on behavioral intention. Two models were tested: Model 1, which includes the core variables of the TPB, and Model 2, which represents the expanded TPB model by adding health literacy and perceived healthcare discrimination. In Model 1 (TPB), the results indicate that attitude (β = 0.231, *p* < 0.001), subjective norms (β = 0.258, *p* < 0.001), and PBC (β = 0.232, *p* < 0.001) all have positive and significant effects on behavioral intention. The model is statistically significant (F = 68.159, *p* < 0.001) and explains 49.7% of the variance in behavioral intention (R^2^ = 0.497). These findings support the assumptions of the TPB, indicating that favorable attitude, stronger social influence, and greater perceived control increase women’s intentions to engage in preventive health behaviors. Accordingly, H1 (attitude → behavioral intention), H2 (subjective norms → behavioral intention), and H3 (PBC → behavioral intention) are supported in Model 1.

In Model 2 (Expanded TPB), the inclusion of additional variables improves the explanatory power of the model. The results show that attitude (β = 0.168, *p* < 0.001), subjective norms (β = 0.215, *p* < 0.001), PBC (β = 0.200, *p* < 0.001), and health literacy (β = 0.302, *p* < 0.001) all have significant positive effects on behavioral intention. Among these variables, health literacy exhibits the strongest influence, highlighting the importance of health-related knowledge in shaping preventive health intentions among women in rural communities. Thus, H4 (health literacy → behavioral intention) is supported, as health literacy demonstrates a strong and statistically significant positive effect. In contrast, perceived healthcare discrimination (β = −0.048, *p* = 0.177) does not show a significant effect on behavioral intention; therefore, H5 (perceived healthcare discrimination → behavioral intention) is not supported in this study. The expanded model is also statistically significant (F = 56.268, *p* < 0.001) and explains 57.5% of the variance in behavioral intention (R^2^ = 0.575), indicating an improvement in explanatory power compared to Model 1. Overall, the results confirm that the extended TPB model provides a better explanation of behavioral intention, with four out of the five proposed hypotheses (H1–H4) supported, while H5 is not supported.

Regarding diagnostic tests, the “Variance Inflation Factor” (VIF) values range from 1.019 to 1.495, and the tolerance values range from 0.669 to 0.982, which are within the recommended thresholds (VIF < 5 and tolerance > 0.20), suggesting that multicollinearity is not a concern [64]. Furthermore, the Durbin–Watson statistics (1.801 and 1.878) fall within the acceptable range, indicating no serious autocorrelation in the residuals.

## 5. Discussion, Implications, and Limitations

### 5.1. Discussion

This study aimed to examine the factors influencing intentions to engage in preventive health behaviors among women in rural communities. The findings revealed that positive attitudes were positively associated with women’s behavioral intentions to engage in preventive health practices. This finding is consistent with the assumptions of the TPB [20]. Huang et al. [25] explained that perceived susceptibility to illness and perceived benefits are associated with more favorable attitudes toward preventive health behaviors. Similarly, Park and Oh [27] found that perceived severity of disease is linked to adolescents’ attitudes toward preventive health behaviors. Moreover, the quality of interaction between patients and healthcare providers, as well as access to reliable sources of information, can strengthen positive attitudes or transform negative ones [8,19,47]. These findings are consistent with prior research demonstrating that attitudes are significantly related to behavioral intentions (e.g., [24,25,26,28,42]). Conversely, negative attitudes, often resulting from dissatisfaction with previous healthcare experiences or exposure to misinformation, may hinder the adoption of preventive health behaviors [3,49]. By addressing misconceptions and providing real-life examples and credible information, health interventions can transform attitudes from hesitation or skepticism into enthusiasm and commitment [10,46,50]. Such a transformation is essential, as positive attitudes not only enhance behavioral intentions but also form the foundation for actual behavioral engagement in preventive health practices [4,21].

In addition, the results indicated that subjective norms are significantly associated with women’s intentions to engage in preventive health behaviors. This highlights the potential influence of social pressure and trusted figures on women’s opinions and behaviors regarding health practices [23]. These findings are consistent with several previous studies showing that subjective norms represent an important predictor of behavioral intentions (e.g., [23,24,26,28]). For example, family support, peer endorsement, the influence of celebrities, or advice from a fitness trainer may play an important role in shaping women’s positive intentions to engage in physical activity as a preventive health behavior [8]. Conversely, this motivation may weaken if such behaviors are met with rejection or social disapproval. In this regard, Fava et al. [47] indicated that subjective norms may negatively influence preventive health behaviors when close relatives, such as husbands or children, overly reassure women with excessive optimism, thereby discouraging them from seeking preventive medical care. So, by fostering a cultural environment that encourages and reinforces preventive health practices, women in rural communities may feel more empowered to seek healthcare services and adopt healthier behaviors [6,21,42].

The results also show that PBC is positively associated with women’s intentions to adopt preventive health behaviors. This suggests that empowering women in rural communities to implement preventive practices enhances their willingness to engage in such behaviors. These findings are consistent with several previous studies showing that PBC is a crucial determinant of preventive health intentions and behaviors [21,23,24,28,51]. Similarly, Hüsser et al. [26] demonstrated that PBC strengthens preventive behavioral intentions in the context of the COVID-19 pandemic. Furthermore, Ausserhofer et al. [8] found that a sense of control and confidence represents one of the key motivators of preventive health behavior. As noted by Satoh and Sato [19], economic factors may constitute a barrier to the adoption of preventive health practices. This barrier may be particularly evident among low-income individuals living in remote areas, which is the case for many women in rural communities with limited financial resources. In practice, some women in rural communities may encounter difficulties in implementing preventive behaviors due to poverty, limited resources, illiteracy, lack of knowledge, and long distances from healthcare centers, among other factors [6,7]. While some of these barriers can be addressed, structural constraints may require broader economic and social policies, such as those related to improving financial resources to support healthier dietary patterns [49]. Therefore, support initiatives should go beyond providing technical resources (to bridge the skills gap) and informational resources (to bridge the knowledge gap) to also include financial support and adequate infrastructure [4,46], which are essential for removing barriers that may prevent women in rural communities from adopting preventive health solutions.

Furthermore, health literacy is found to be strongly and positively associated with women’s intentions to adopt preventive health behaviors, highlighting the important role of health knowledge in disease prevention and in strengthening women’s health awareness. These findings are consistent with previous empirical studies demonstrating a positive relationship between levels of health literacy and preventive health behaviors [1,2,8,19,29,31,45,52]. Such studies have reported positive associations between health literacy and preventive health practices, particularly among less-educated women, through the information they receive. Undoubtedly, the lack of formal sources of health information may make women more vulnerable to informal information sources [5,50,53], which may sometimes be distorted or inaccurate, thereby influencing their attitudes and behaviors. In practice, individuals who seek to avoid health risks and problems are more likely to adopt preventive health behaviors [19]. Hence, reliable information explaining potential health risks may serve as a strong motivator for adopting preventive health measures [3,54]. Working women may also be more exposed to health recommendations and campaigns promoting preventive behaviors in the workplace compared with unemployed women or those who are self-employed [19]. Consequently, social marketing strategists should consider these different groups and reach them through appropriate communication channels [15,16]. Nevertheless, Ausserhofer et al. [8] noted that health information alone may not be sufficient to produce changes in preventive health behavior. Interventions designed to improve health literacy, through educational programs and accessible health information can significantly enhance women’s willingness to adopt preventive health behaviors [48]. Therefore, by equipping women with the necessary knowledge and skills to advocate for their own health, it is possible to build a more informed and proactive community capable of adopting preventive health measures.

Finally, a particularly noteworthy finding of this study is the non-significant effect of perceived healthcare discrimination on behavioral intention. Although the relationship was negative, it did not reach statistical significance, suggesting that perceived discrimination may not be a primary factor influencing preventive health intentions among women in rural communities in this context. One possible explanation relates to the specific socio-cultural context of rural Algeria. In relatively homogeneous rural communities, perceptions of discrimination may be less salient or less explicitly recognized compared to more diverse societies. Additionally, strong family support systems and community ties may buffer the potential negative effects of perceived discrimination. Furthermore, limited healthcare alternatives in rural areas may lead women to engage in preventive health behaviors regardless of perceived discrimination, particularly when health concerns are considered important or urgent. Another explanation is that other factors identified in this study, particularly health literacy and PBC, may play a more dominant role in shaping behavioral intentions, thereby reducing the relative influence of perceived discrimination. This suggests that informational and capability-related factors may be more actionable levers for intervention in this context.

Previous studies have reported mixed findings regarding this relationship. Several investigations have shown that perceived healthcare discrimination can negatively affect individuals’ engagement in healthy behaviors and healthcare utilization [35,49,59]. In contrast, Cruz-Riveros et al. [62] identified a positive association, suggesting that experiences of discrimination may sometimes motivate individuals to become more proactive in managing their health, possibly as a coping or adaptive response to perceived adversity. These inconsistent findings indicate that economic, cultural, and social contexts may play an important role in shaping how discrimination influences health-related behaviors. For example, perceptions of discrimination may be more salient in highly diverse societies, such as the United States, where racial, ethnic, and cultural differences are more visible within healthcare systems. In this regard, Klein et al. [59] and Okoro et al. [61] emphasize the importance of addressing discrimination within medical education and professional training programs. Integrating strategies to recognize and reduce discriminatory attitudes can help healthcare professionals provide more equitable care. Furthermore, several scholars highlight the value of incorporating principles of cultural humility into healthcare training curricula, as these approaches encourage healthcare providers to develop greater awareness of cultural differences and implicit biases [38,49,60]. Such initiatives can contribute to reducing structural barriers and health disparities experienced by vulnerable populations, including women in rural communities.

### 5.2. Practical Implications

The findings of this study highlight the important role of the three core constructs of the PBT (attitude, subjective norms, and PBC) in understanding behavioral intentions toward preventive health practices among women in rural communities. Accordingly, it is essential to strengthen positive attitudes among women in rural communities toward preventive health behaviors by clearly communicating the potential risks of neglecting preventive care and the benefits associated with adopting healthy practices. In addition, interventions should address negative attitudes or misconceptions that may arise from previous experiences or inaccurate health information. At the same time, subjective norms should be actively leveraged, particularly through the involvement of community influencers such as local leaders, religious figures (e.g., mosque imams), educators, and respected community members, who can play an important role in promoting a broader culture of health prevention within rural communities. Furthermore, stakeholders should collaborate to reduce the barriers that may hinder women in rural communities from adopting preventive health behaviors.

In addition, the results emphasize the significant role of health literacy in promoting preventive health behaviors. Therefore, health awareness campaigns and educational programs can equip women in rural communities with essential knowledge and skills related to the risks associated with certain diseases, particularly women’s health conditions, and the methods of prevention and early detection. To further strengthen health literacy, health interventions should focus on providing supportive resources, such as transportation services, community-based educational workshops, and accessible health information, enabling women to overcome practical constraints. To maximize the effectiveness of these initiatives, it is important to consider the educational level, age, and socio-economic and psychological conditions of the targeted populations. Such interventions can be implemented through field visits to rural communities, distribution of informational materials, local radio broadcasts, and the organization of regular community meetings. Moreover, multiple stakeholders, including schools, universities, mosques, and health-related associations, can play a vital role in enhancing health literacy within rural communities.

### 5.3. Policy Implications

Based on the findings, this study offers several policy-related recommendations. Although Algeria has made significant efforts to improve healthcare services for all citizens across different regions and continues to work toward enhancing the economic and social conditions of disadvantaged areas, greater attention should be directed toward women in rural communities by designing policies that consider their specific circumstances and needs. For instance, policies aimed at improving the overall economic conditions of women in rural communities could substantially enhance their quality of life and enable them to secure essential resources such as healthy food, clean drinking water, clothing, and adequate bedding. Improving these basic living conditions can indirectly support better health outcomes and encourage the adoption of preventive health behaviors.

At the same time, policymakers may consider implementing targeted health policies, such as launching large-scale screening campaigns to accurately diagnose diseases among women in rural communities and identify their underlying causes. Such initiatives would provide valuable data that can support the development of more effective preventive health programs tailored to the needs of rural populations. As an additional step, the findings of this study may also support the use of social marketing tools to design, implement, and monitor preventive health interventions in rural communities [9,15].

Moreover, digital health policies that specifically address the needs of women in rural communities could play an important role in overcoming barriers to healthcare access, reducing health disparities, and promoting greater equity in healthcare services. Technologies such as telemedicine platforms, mobile health applications, and digital awareness programs can expand access to medical information and services, particularly in geographically remote areas.

In summary, various stakeholders, including health authorities, policymakers, and community organizations, can benefit from the findings of this study to design more effective policies and interventions aimed at improving healthcare outcomes and promoting preventive health behaviors among women in rural communities.

### 5.4. Limitations and Future Directions

Despite its contributions, this study has several limitations that should be acknowledged. First, the study relied on a convenience sampling method and focused exclusively on women in rural communities, which may limit the generalizability of the findings to other regions or population groups. In addition, the sample was largely facility-based, as participants were recruited from healthcare-related settings such as clinics, pharmacies, and screening centers. This may have introduced a selection bias toward women who are already connected to or engaged with healthcare services, and who may therefore exhibit higher levels of health awareness and preventive intentions compared to the broader rural population. Therefore, future research could expand the sample to include participants from other geographical regions in Algeria, such as the southern areas, as well as urban populations, allowing for comparative analyses and a better understanding of potential contextual differences. Second, the data were collected through self-administered questionnaires, which may be subject to response bias or social desirability effects. Future studies could address this limitation by employing mixed-method approaches, combining quantitative surveys with qualitative techniques (such as interviews or observations) to capture a more comprehensive picture of actual preventive health behaviors. Third, the cross-sectional design of the study limits the ability to draw causal inferences between variables. The findings should therefore be interpreted as associations rather than cause-and-effect relationships. Future research could adopt longitudinal or experimental designs to better examine causal pathways and changes in preventive health behaviors over time. Fourth, although the extended model explained 57.5% of the variance in women’s intentions to adopt preventive health behaviors, future research could further enrich the model by incorporating additional relevant variables, such as access to healthcare services, cultural beliefs, trust in healthcare providers, or digital health literacy. In addition, future research could extend the present model by incorporating key socio-demographic variables (e.g., age, education level, marital status, occupation, and income) as control, mediating, or moderating factors, in order to better capture the role of cultural and structural differences in shaping health literacy and preventive health intentions among women in rural communities. Fifth, building on the present findings, future research could also investigate the effectiveness of specific intervention strategies, such as community-based health programs, outreach initiatives, or digital health solutions, in improving preventive health behaviors among women in rural communities. Finally, future studies could benefit from integrating other theoretical perspectives or combining frameworks, such as the HBM, “Social Cognitive Theory” (SCT), or the Information Adoption Model, to provide a deeper understanding of preventive health behaviors.

## 6. Conclusions

This study contributes to the growing body of research on preventive health behaviors among women in rural communities by applying and extending the TPB. Consistent with the study objective of examining the factors associated with behavioral intentions, the findings indicate that attitude, subjective norms, and PBC are significantly associated with intentions to adopt preventive health behaviors among women in rural communities. These results support the explanatory relevance of the TPB framework in this context. In addition, health literacy emerges as a strong correlate of behavioral intention, suggesting that women with higher levels of health-related knowledge and understanding tend to report stronger intentions to engage in preventive practices. In contrast, perceived healthcare discrimination does not show a statistically significant association with behavioral intention, highlighting the context-dependent nature of this factor. Overall, the extended model explains a substantial proportion of variance in behavioral intention, indicating the importance of both psychosocial and informational factors in understanding preventive health intentions among women in rural communities. However, these findings should be interpreted as associations rather than causal relationships, and they relate to intentions rather than actual health behaviors.

From a practical and policy perspective, the results suggest that public health practitioners and policymakers working in rural settings may benefit from focusing on strategies that enhance positive attitudes toward preventive health, strengthen supportive social environments, improve women’s perceived ability to engage in preventive practices, and promote access to reliable and understandable health information. By aligning health promotion efforts with these factors, stakeholders can better support engagement with preventive health practices among women in rural communitiesand contribute to efforts aimed at reducing health disparities and improving public health outcomes in underserved communities.

## Figures and Tables

**Table 1:** Sample characteristics (*N* = 205).

Variable	Description	*n*	%
Marital status	Single	63	30.73
	Married	104	50.73
	Divorced	11	5.37
	Prefer not to say	27	13.17
Age	20–30 years	42	20.49
	31–40 years	70	34.15
	41–50 years	57	27.80
	51–60 years	36	17.56
Education	High school or below	81	39.51
	Bachelor’s degree	66	32.20
	Master’s degree	45	21.95
	Doctorate	13	6.34
Occupation	Student	38	18.54
	Employed (public or private sector)	65	31.71
	Self-employed	40	19.51
	Unemployed	62	30.24

**Table 2:** Descriptive Statistics.

Constructs	Mean	Std.	Alpha	Skewness	Kurtosis
1. AT	3.56	0.65	0.899	−0.443	−0.150
2. SNs	2.82	0.60	0.881	0.276	−0.155
3. PBC	3.69	0.74	0.875	−0.688	0.285
4. HL	4.27	0.52	0.701	−1.419	3.625
5. PHD	3.14	0.65	0.762	−0.141	−0.583
6. BI	3.99	0.50	0.711	−0.237	−0.261

Note: Subjective norms (SNs), perceived behavioral control (PBC), health literacy (HL), perceived healthcare discrimination (PHD), behavioral intention (BI).

**Table 3:** Construct correlations.

Constructs	1	2	3	4	5	6
1. AT	1					
2. SNs	0.201 **	1				
3. PBC	0.536 **	0.259 **	1			
4. HL	0.380 **	0.281 **	0.348 **	1		
5. PHD	0.022	0.117	0.080	0.066	1	
6. BI	0.545 **	0.459 **	0.582 **	0.568 **	0.017	1

Note: Subjective norms (SNs), perceived behavioral control (PBC), health literacy (HL), perceived healthcare discrimination (PHD), behavioral intention (BI). **. Correlation is significant at the 0.01 level (2-tailed).

**Table 4:** Hierarchical Regress Analysis.

Constructs	β	t-Value	Sig.	Tolerance	VIF
(constant)	1.582	9.210	0.000		
Attitude	0.231	5.082	0.000	0.708	1.412
SNs	0.258	6.017	0.000	0.928	1.078
PBC	0.232	5.701	0.000	0.689	1.452
Model 1 (TPB): F = 68.159 (*p* < 0.001); R^2^ = 49.7%; Durbin-Watson = 1.801					
(constant)	0.912	4.041	0.000		
Attitude	0.168	3.895	0.000	0.669	1.495
SNs	0.215	5.312	0.000	0.883	1.132
PBC	0.200	5.287	0.000	0.671	1.490
HL	0.302	6.142	0.000	0.793	1.260
PHD	−0.048	−1.355	0.177	0.982	1.019
Model 2 (Expanded TPB) F = 56.268 (*p* < 0.001); R^2^ = 57.50%; Durbin-Watson = 1.878					

Note: Subjective norms (SNs), perceived behavioral control (PBC), health literacy (HL), perceived healthcare discrimination (PHD). Source(s): Authors’ own work.

## Data Availability

The original contributions presented in this study are included in the article. Further inquiries can be directed to the corresponding author.

## Appendix A

**Table A1:** Measurement items.

Variable	Items	Sources
Attitudes toward the behavior (AT)	AT1. Engaging in preventive health behaviors (e.g., regular check-ups, vaccinations, and health screenings) is beneficial for my health.	Bouarar et al. [12] and Huang et al. [25]
	AT2. Practicing preventive health behaviors is a wise decision for maintaining good health.	
	AT3. Overall, adopting preventive health behaviors is important for women like me.	
Subjective norms (SN)	SN1. People who are important to me think that I should engage in preventive health behaviors.	Brouwer and Mosack [63] and Park and Oh [27]
	SN2. My family encourages me to practice preventive health behaviors.	
	SN3. Women in my community believe that preventive health behaviors are important.	
Perceived behavioral control (PBC)	PBC1. I feel confident in my ability to adopt preventive health behaviors.	Hüsser et al. [26]
	PBC2. If I wanted to, I could easily engage in preventive health practices.	
	PBC3. I have the necessary resources (time, transportation, or support) to practice preventive health behaviors.	
Health literacy (HL)	HL1. I can easily understand health information related to disease prevention.	Ausserhofer et al. [8]
	HL2. I know where to find reliable information about preventing diseases.	
	HL3. I feel capable of making informed decisions about my health based on available information.	
Perceived healthcare discrimination (PHD)	PHD1. I feel that some women are treated unfairly in healthcare facilities because of their background or social status.	Hausmann et al. [40] and Krieger et al. [41]
	PHD2. Healthcare providers sometimes treat rural women with less respect than others.	
	PHD3. Fear of unfair treatment in healthcare facilities discourages some women from seeking care.	
Behavioral Intention (BI)	BI1. I intend to engage in preventive health behaviors in the near future.	Brouwer and Mosack [63] and Hüsser et al. [26]
	BI2. I plan to regularly participate in preventive health practices (such as check-ups or screenings).	
	BI3. I will make an effort to follow preventive health recommendations.	
